# Integration of a 2D Touch Sensor with an Electroluminescent Display by Using a Screen-Printing Technology on Textile Substrate

**DOI:** 10.3390/s18103313

**Published:** 2018-10-02

**Authors:** Josue Ferri, Clara Perez Fuster, Raúl Llinares Llopis, Jorge Moreno, Eduardo Garcia‑Breijo

**Affiliations:** 1 Textile Research Institute (AITEX), 03801 Alicante, Spain; josue.ferri@aitex.es (J.F.); jmoreno@aitex.es (J.M.); 2 Departamento de Comunicaciones, Universitat Politècnica de València, 03801 Alcoy, Spain; rllinares@dcom.upv.es; 3 Instituto Interuniversitario de Investigación de Reconocimiento Molecular y Desarrollo Tecnológico (IDM), Universitat Politècnica de València, Universitat de València, 46022 Valencia, Spain; cperezf@eln.upv.es

**Keywords:** 2D touchpad, electroluminescent, wearable sensing, textile, screen-printing technology

## Abstract

Many types of solutions have been studied and developed in order to give the user feedback when using touchpads, buttons, or keyboards in textile industry. Their application on textiles could allow a wide range of applications in the field of medicine, sports or the automotive industry. In this work, we introduce a novel solution that combines a 2D touchpad with an electroluminescent display (ELD). This approach physically has two circuits over a flexible textile substrate using the screen-printing technique for wearable electronics applications. Screen-printing technology is widely used in the textile industry and does not require heavy investments. For the proposed solution, different layer structures are presented, considering several fabric materials and inks, to obtain the best results.

## 1. Introduction

To improve the effectiveness of interfaces such as touchpads, buttons, or keyboards, a feedback mechanism is necessary to confirm to the user that the order or action has been recognized by the system. Many types of solutions have been studied and developed in order to provide this user feedback. In this sense, there exist solutions based on haptic feedback by vibrating the touchpad surface [1]. This type of solution has been applied in the automotive industry to assess its effectiveness in real driving situations [2]. On the other hand, the use of tiny sounds used as feedback has also been studied to inform the user that the action has been recognized in combination with or without vibration systems [3,4]. For blind people, different approaches have been studied, such as small braille mechanisms integrated under the touchpad, which emerge as the user interacts with the system [5]. Regarding touchpads, different technologies have been developed as solutions for keyboards, touch panels [6], and touchscreens [7]. From all of them, capacitive and resistive touch technologies master the touch landscape now [8]. Other solutions have been investigated, such as optical sensors [9], force sensors [10], or even inductive sensors [11]. In addition, flexible and stretch sensing approaches have also been popular using contact resistances between threads [12,13], with piezoelectric [14] and capacitive designs [15,16,17].

Recently, there has been a considerable growing interest in the development of new interfaces that could be printed onto, embedded or attached into textile, giving them a wearable new feature. These interfaces should be flexible, comfortable, and cheap to be suitable for integration into textiles that become smart textiles, fabrics, or garments. The application of these interfaces is very promising with possibilities of interaction with computers, smartphones, or Internet of Things (IoT) devices. In a recent work, our team has designed and developed different touchpad prototypes based on capacitive (pro-cap) technologies using textile substrates [6]. The technique used in these touchpads, screen-printing, is low cost and quite habitual in the textile printing industry.

On the other hand, in spite of the fact that the electroluminescent lamps are not a new technology, electroluminescent display (ELDs) have seen an increase in interest lately due to smart fabrics. Flexible displays have experienced a significant development in recent years, with interest being driven by the expanding wearable technology market. Several experiments using different materials for the different layers, especially for the phosphor and transparent conductive layers, have been performed characterizing the material properties [18]. Other approaches consider including flexible substrates as fabrics evaluating flexibility and breathability [19]. Also, complex seven-segment digital displays have been developed using screen-printing electroluminescent printed on fabric substrates [20].

There are different alternatives to electroluminescent ones like displays based on electrochromic materials [21,22]. Thus, fiber-based visualizers [21] and those based on electrochromic tissues [23] have been reported. The new OLED (Organic Light Emission Diode) technologies still have little implementation in textiles but some developments have already been reported [22,24,25,26]. Finally, polymeric optical fiber fabrics have also been used for illumination and sensorial applications in textiles [27,28].

Yet, to date, there have been only limited papers on solutions combining simple interfaces with light emission as feedback. Some solutions are based on flexible and stretchable substrates, such as Poly(dimethylsiloxane) (PDMS), combined with an ELD [29]. This innovative solution can be used as a button or keyboard that is illuminated, as soon as the user pulsation is detected. Other authors address its application on paper and textiles as substrates with the use of capacitive buttons [30].

In this work, we introduce a novel solution that combines complex interface, a touchpad 2D with an electroluminescent ELD. This approach physically has two circuits over a flexible textile substrate using the screen-printing technique for wearable electronics applications. In addition, different layer structures are presented, considering different fabric materials and inks, to obtain the best results.

## 2. Materials and Methods

### 2.1. Device Architecture Development

**Figure 1:**
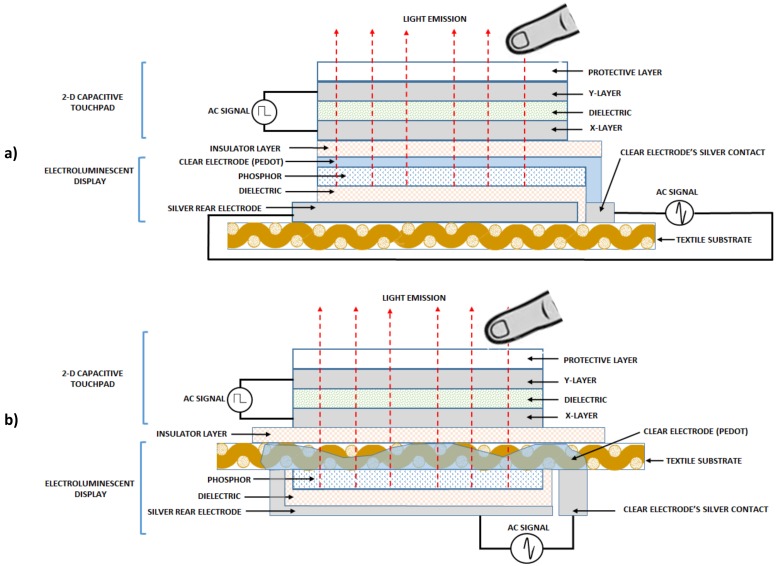
ELD + 2D touchpad architecture: All the design on one side of the textile (**a**); Using the textile itself as a separating element, on one side the ELD and on the other one the 2D touchpad sensor (**b**). ELD: electroluminescent display.

The architecture of the device consists of, on the one hand, a 2D touchpad whose design is similar to one developed by Ferri, J. et al. [6], and on the other hand, an ELD whose classic design has two conductive external electrodes [31]. In order to manufacture the 2D touchpad sensor in combination with the display, one of the conductive electrodes of ELD must be isolated, specifically the emitting electrode. Two designs can be used for the device:

- Using the fabric as a base, silk-screen printing is done, first corresponding to the ELD and next, corresponding to the 2D sensor. An insulator must be inserted between the two layers (Figure 1a).
- Using the own fabric as part of the emitting electrode, the different layers of the electroluminescent are printed in the lower part of the fabric and the 2D sensor in the upper part. This upper part is conveniently insulated in those cases in which the conductive material of the emitting electrode is completely embedded in the fabric (Figure 1b).

The second structure has been chosen for this work, since the fabric can be used as a support for the transparent conductor and at the same time as an insulating layer, saving layers in the process. The development of each of the elements, the ELD on the one hand and the 2D on the other one, are detailed below.

#### 2.1.1. Electroluminescent Display Development

**Figure A1:**
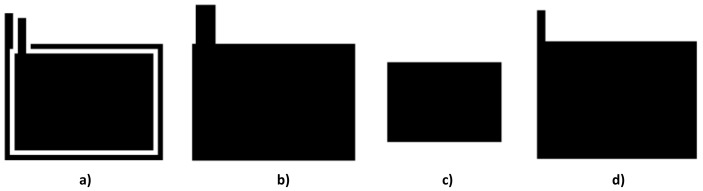
ELD Design: Conductive silver electrode (**a**); dielectric layer (**b**); Phosphor layer (**c**); and clear conductor (**d**).

There are two standard configurations for a printed ELD, also named ACPEL (AC Powder Electroluminescent) [32]. In the first one, the light is emitted through a transparent substrate, whereas in the second one, the light is emitted through a printed transparent conductor [33]. To avoid the use of a transparent conductive film (Indium Tin Oxide (ITO) type) required by the first configuration, the second option has been used in this work. Four layers are needed to build the ELD; they are shown in Appendix A, Figure A1: rear conductor layer (a); dielectric layer (b); phosphor layer (c); and clear conductive layer (d).

Thus, to build the ELD, four screens were made. The screen for the conductors (Appendix A, Figure A1a,d), Silver and PEDOT:PSS, was a 230 mesh polyester material (PET 1500 90/230-48 from Sefar, Thal, Suiza) and the screen for the Dielectric and Phosphor layers (Appendix A, Figure A1b,c), was a 175 mesh polyester material (PET 1500 68/175-64 PW from Sefar). Afterwards, to transfer the stencil to a screen mesh, a Dirasol 132 (Fujifilm, Minato, Japan) UV film was used. The final screen thicknesses were 10 μm for the screen for the conductors and 15 μm for the screen for the dielectric and phosphor. The patterns were transferred to the screen by using a UV light source unit.

The inks used were C2131014D3 Silver paste (Gwent Group, Pontypool, UK) as rear electrode layer, D2070209P6 White Dielectric Ink (Gwent Group) as dielectric layer, C2070126P5 White Phosphor Ink (Gwent Group) as phosphor layer and C2100629D1 Clear conductor ink (Gwent Group) as emitting electrode. Flexibility is one of the most important characteristics of these inks to use them with textiles.

Printing was carried out by using an Ekra E2 XL screen-printer with a 75° shore squeegee hardness, 3.5 bar force, and 8 mm/s. After inks depositing, these were cured in an air oven (MEMMERT UNB-100) at 130 °C for 10 min.

**Figure 2:**
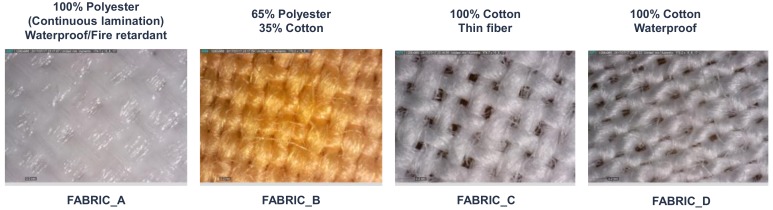
Different types of fabrics used as substrates.

Several materials were used for the substrate: Mediatex TT ACQ 120 μm (Technohard, Barcelona, Spain) 100% polyester (Fabric_A), a mix of 65% polyester–35% cotton (Fabric_B), a 100% cotton (Fabric_C), a 100% cotton waterproof (Fabric_D). In order to compare the color, luminosity, and transmission of the textiles, two totally transparent substrates were tested as well: an ITO film and a transparent polyurethane (Inspire^®^ 2370, Coveris^TM^ Advanced Coatings, Matthews, NC, USA). In the case of the 75 µm ITO/PET film F2071018D1 (Gwent Group), the clear conductor layer was the own ITO. Figure 2 shows the substrates used and their characteristics are shown in Appendix A, Table A1.

In order to improve the response of the clear conductor ink, two compounds were used: Triton™ X-100 (Sigma-Aldrich, San Luis, MO, USA) as nonionic surfactant to improve the wettability and Glycerol (Sigma-Aldrich, San Luis, MO, USA) to improve the mobility [34,35]. The behavior of the PEDOT:PSS has been studied in the natural fibers, which allow an absorption of this material (Fabric_B and Fabric_C), not so the Fabric A and D samples since the Fabric_A is 100% waterproof polyester and the Fabric_D is cotton but with a water-resistant treatment. The PEDOT:PSS was tested alone and adding the two aforementioned compounds: Triton™ X-100 (Sigma-Aldrich, San Luis, MO, USA) for wettability and Glycerol (Sigma-Aldrich, San Luis, MO, USA) for mobility. Therefore, three more samples have been studied: Fabric_C + Triton (2.0 wt %), Fabric_C + Glycerol (10 wt %) and Fabric_C + Triton + Glycerol.

2.1.2. 2D Touchpad Development

**Figure A2:**
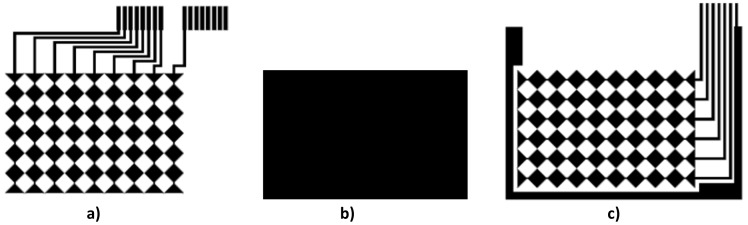
2D Touchpad Design: Vertical or X layer (**a**); dielectric layer (**b**); and Horizontal or Y layer (**c**).

A sensor matrix formed by 9 × 6 electrodes has been designed. The sensor has been developed with two conductive layers for horizontal and vertical tracks and another layer of dielectric. The three patterns are shown in the Appendix A, Figure A2: Vertical or X layer (a); dielectric layer (b); and Horizontal or Y layer (c). The Pitch (Row and Column) of 8 mm and Gap of 0.4 mm are the main dimensions of pattern.

To build the sensor matrices, three screens were made. The screen for the conductors (Appendix A, Figure A2a,c) was a 230 mesh polyester material (PET 1500 90/230-48 from Sefar) and the screen for the dielectric layer (Appendix A, Figure A2b) was a 175 mesh polyester material (PET 1500 68/175-64 PW from Sefar). Afterwards, to transfer the stencil to a screen mesh, a Dirasol 132 (Fujifilm) UV film was used. The final screen thicknesses were 10 μm for the screen for the conductors and 15 μm for the screen for the dielectric. The patterns were transferred to the screen by using a UV light source unit.

The inks used were C2131014D3 Silver paste (Gwent Group) as conductive layers, D2081009D6 Transparent Green Colored ink (Gwent Group) as dielectric layer. Two layers of dielectric are needed to improve the design as it is explained in Ferri, J. et al. [6].

Printing was carried out by using an Ekra E2 XL screen-printer with a 75° shore squeegee hardness, 3.5 bar force, and 8 mm/s. After the deposition of the inks, these were cured in an air oven (MEMMERT UNB-100) at 130 °C for 10 min.

Substrates are the same detailed in the previous point.

### 2.2. Electronic Systems Development

**Figure 3:**
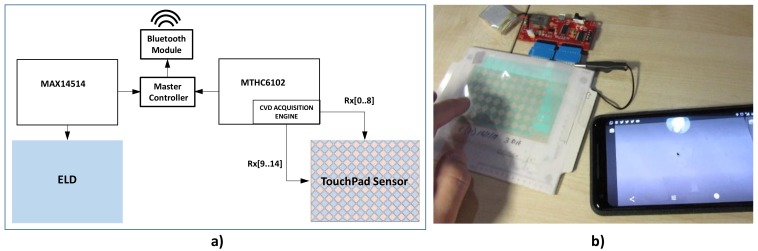
Electronic System Block Diagram (**a**); real electronic system applied to a mouse control in a mobile phone (**b**).

The electronic systems consist of four blocks. A master controller, implemented with a PIC16LF1454 that controls other two blocks: one for the ELD and the other one for the 2D touchpad (Figure 3). The last block corresponds to a Bluetooth module to make the system portable. Figure 3a shows the complete system with the two control blocks, one for the ELD and one for the touchpad, and the master block, with the different integrated circuits in each block. Figure 3b shows an application of the system implementing a mouse for a mobile phone through a Bluetooth communication.

#### 2.2.1. ELD Electronic Block

The ELDs are a parallel-plate “lossy capacitors”, an active electroluminescent phosphor is embedded in dielectric. The application of an AC voltage to both plates generates a changing field within the active layer that causes the phosphor to emit light.

Electroluminescent displays require special frequency, voltage and waves types characteristics. Thus, for its excitation it is necessary to apply a sinusoidal or square alternating signal of amplitude between 100 to 400 V and of frequency of the order of 50 to 900 Hz.

In order to use stand-alone power sources (batteries), an electronic circuit is needed which, from a direct voltage, provides us with the alternating signal required by the electroluminescent lamp. The integrated circuit MAX14514 (Maxim Integrated) has been selected. The MAX14514 features a +2.7 V to +5.5 V input range that allows the device to accept a wide variety of voltage sources, including single-cell lithium-ion (Li+) batteries. The lamp outputs of the device generate up to 300 V_P-P_ for maximum lamp brightness.

#### 2.2.2. 2D Touchpad Electronic Block

MTCH6102 (Microchip) has been used in this work. This device is a turnkey projected capacitive touch controller that simplifies adding gestures to touch interface designs with industry-leading low-power performance. It utilizes up to 15 channels to support taps, swipes, and scrolling on XY touch pads and touch screens. The operation and the scheme are explained and showed in Ferri, J. et al. [6].

## 3. Results and Discussion

### 3.1. Physical Parameters

**Figure 4:**
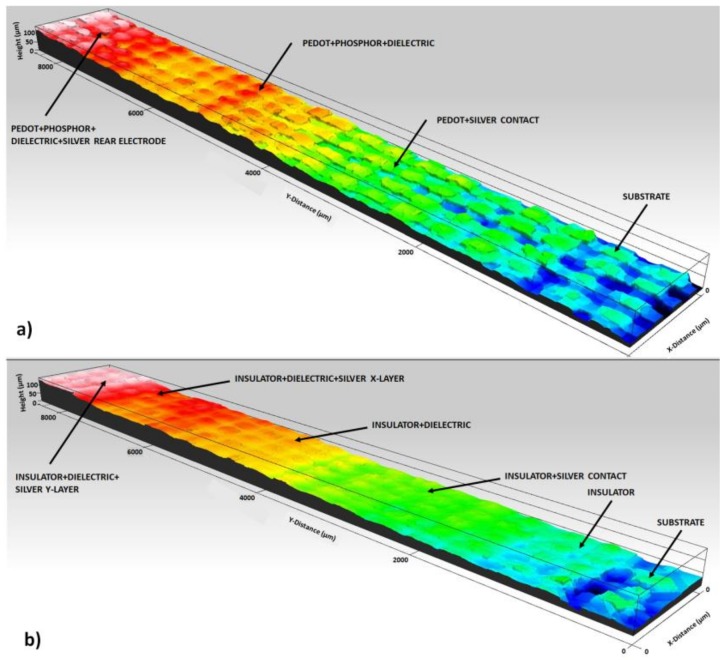
Electroluminescent Display layers profilometry (**a**). 2D Touchpad layers profilometry, in this case fabrics A and B are studied in order to value the insulator layer (**b**).

The profilometer Profilm3D (Filmetrics) has been used to measure the thickness of the set of layers on each side of the fabric. Figure 4 shows the profilometry of the two faces of the fabric. In Figure 4a, the ELD layers with a final average thickness of 70 μm are shown for the Fabric_A substrate, distributed in approximately 3 μm of PEDOT:PSS, 26 μm of phosphorus, 35 μm of dielectric and 6 μm of silver. In this type of substrate, the thickness of the PEDOT:PSS layer can be measured since it does not penetrate the fabric, in the rest of the substrates with cotton part of the PEDOT:PSS remains embedded in the fabric. Figure 4b shows the layers of the 2D touchpad, in this case for a Fabric_B type substrate to assess the influence of the insulating layer. The resulting average thickness is 80 μm, distributed in approximately 30 μm of insulation, 9 μm of silver X-layer, 31 μm of dielectric and 10 μm of silver Y-layer.

**Figure A3:**
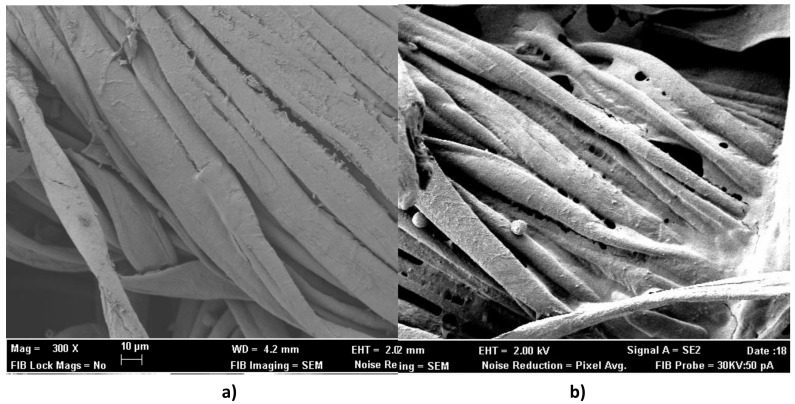
Cotton fibers without PEDOT:PSS (**a**) and after the screen printing of PEDOT:PSS (**b**).

In the Fabric_B and Fabric_C samples, the PEDOT:PSS is partially embedded inside the threads but it covers its surface. Scanning electron microscopy (SEM) images (JEOL JSM6300) have been carried out to verify how the PEDOT interacts with the fibers (Appendix A, Figure A3). In Appendix A, Figure A3a, the cotton fabric without PEDOT:PSS is shown and in Appendix A, Figure A3b the cotton fabric with PEDOT:PSS is shown. A comparison between two images reveals that a PEDOT:PSS coating of the fibers takes place and, in addition, PEDOT:PSS intertwines with the fibers like a ligament [36].

**Figure 5:**
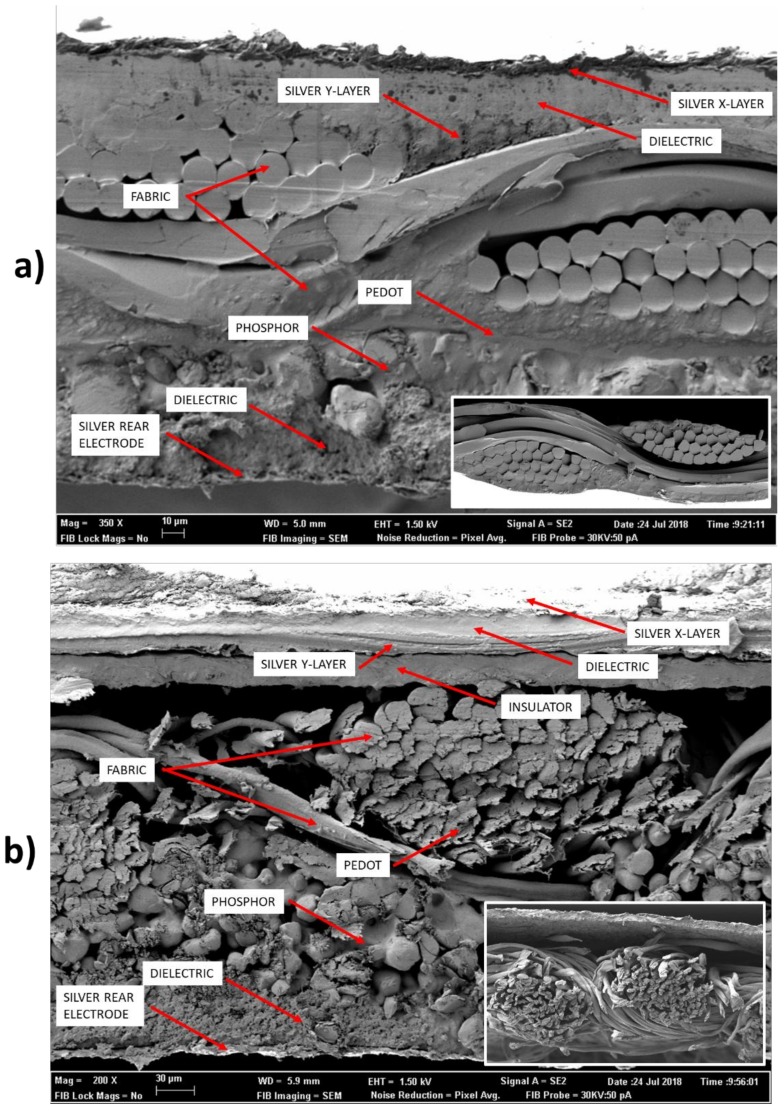
SEM micrograph showing device cross-section. Fabric_A (**a**) and Fabric_C (**b**). In a box, in the bottom right corner of each figure, the virgin fabric is shown.

Finally, a SEM micrograph cross-section of the whole system is shown in Figure 5 to highlight the multilayer structure of the device. Figure 5a shows the Fabric_A sample, the touchpad is on the upper part of the fabric and the ELD on the bottom. In a box, in the bottom right corner, the virgin fabric 100% polyester is shown. Figure 5b shows the Fabric_C sample, on the upper part of the fabric is the touchpad and on the lower part the ELD. In a box, in the bottom right corner, the 100% cotton fabric is shown virgin. In this case, the insulator layer can be observed. This layer avoids the contact between the cotton with PEDOT:PSS and the Silver X-Layer. Moreover, this layer allows to soften the uneven surface of the woven fabric to provide a more heterogeneous surface for the subsequent layers to be printed.

### 3.2. Electroluminescent Display Results

The ELD has been tested using four devices, a programmable AC power source (Chroma Programmable AC Source Model 61601, Taoyuan City, Taiwan) that allows supplying output voltage from 0 to 300 VAC, and frequencies from 15 to 1000 Hz, a fiber-based spectrometer (Thorlabs Compact Spectrometer CC5200/M, Newton, NJ, USA) with a wave range between 350 and 70 nm, and a digital luxometer, a device for precise light measurements of up to 200,000 lux (Koban Digital Lux Meter KL1330, Siero, Spain). The light transmission has been measured with an Instrument System Model Digilux 9500 system photometer in base on the standard NF P38-511:1969.

**Figure 6:**
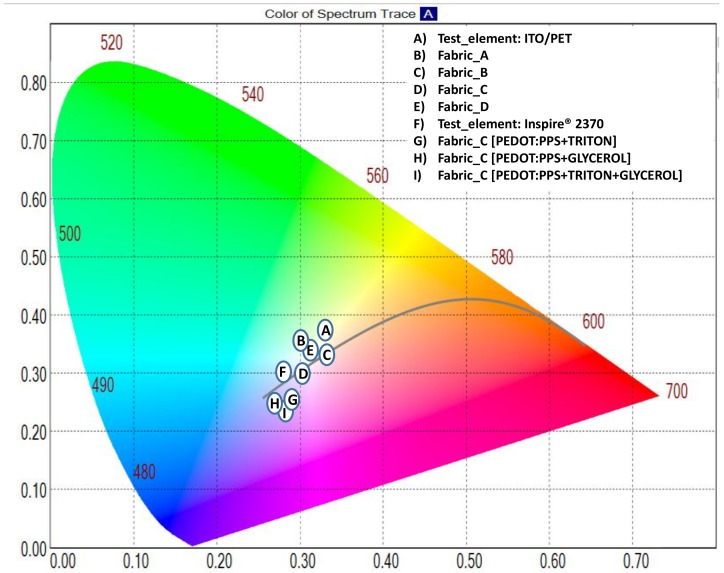
Chromaticity diagram according to the CIE 1931 standard.

To measure the ELD, the ink colours will be defined based on the CIE colour system. The CIE colour system provides a quantitative link between distributions of wavelengths in the electromagnetic visible spectrum, and physiologically perceived colours in human vision. The different displays have been tested at the same conditions applying typical values of 100 V AC and frequency of 400 Hz. Notice that brightness of the display can be increased by a higher voltage and frequency, however both these will shorten the life of the display. The results of the chromaticity, luminance, and light transmission are presented in Figure 6 and Appendix A, Table A2. Although the phosphor ink is the same and has been applied with the same procedure, there are variations in the colour, mainly due to the fabric. A totally transparent test sample (A) is used as a basis of comparison. Fabric_A (B), Fabric_B (C), Fabric_C (D), and Fabric_D (E) are close to the test sample, and with very similar parameters in colour but significantly smaller in order of luminance (three times smaller in the best case (B)) due to the fabric tissue absorbance. No significant improvements have been achieved in the substrates in which an attempt to improve the PEDOT:PSS response has been made (G, H, and I), chromaticity and luminance have not been affected.

### 3.3. 2D Touchpad Results

**Figure 7:**
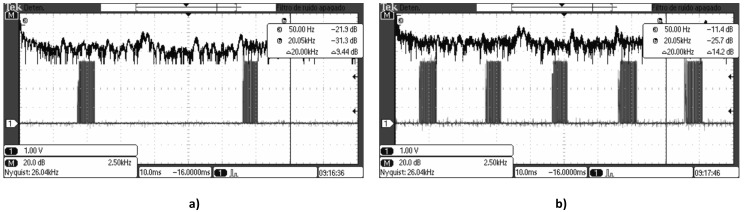
Train of pulses sent by the Touch Controller. Normal signal (**a**) and disturbed signal (**b**).

The operation of the system is explained in Ferri, J. et al. [6]. The touch controller used, MTCH6102, transmits a train of square pulses of 50 µs (20 kHz) of duration with a frequency of 50 Hz. Figure 7a shows the typical signal emitted by the MTCH6102.

The touchpad works correctly when the ELD is switched off, but a problem is detected when turning on the ELD. The ELD produces electromagnetic interferences (EMI) that distort the signal in the touchpad. The signal affected by the emissions is shown in Figure 7b; in the spectrum the 50 Hz frequency suffers a degradation of 10 dB and the 20 kHz frequency decreases 6 dB. Different waveforms, voltage levels, and working frequencies have been studied but an efficient reduction of the emission is not achieved. Therefore an EMI isolation is needed.

An EMI shielding limits the penetration of electromagnetic fields into a space, by blocking them with a barrier made of a conductive material [37]. Different materials can be used as EMI shielding as Al/Cu foil tape, silver paint, copper paint, nickel paint, metal cables, or conductive elastomers depending on the applications [38,39,40]. To isolate the 2D touchpad, an EMI shield must be inserted between the ELD and the 2D Touchpad. The premise is that the EMI shield must be transparent. For this reason an ITO film, which is transparent to visible light but still electrically conducting at the frequencies of interest for EMI shielding (GHz to DC), has been used. Although indium tin oxide (ITO) films have been extensively used in electronic and photoelectronic applications because of their low electrical resistivity, there are few publications discussing the electromagnetic shielding effectiveness of this material. Nevertheless, according to different studies, they can be used as electromagnetic shields [41,42]. The ITO attenuation in different frequencies have been tested, the fewer frequency applied, the more attenuation obtained. If the surface resistance is around 80 Ω/sq, the attenuation at 200 Hz can be around 150 dB [42].

The film used is a 50 µm printed ITO (Multek, Hong Kong, China) and has been fixed to the fabric with a screen-printed adhesive SILPURAN^®^ 2114 A/B (Wacker, Munich, Germany).

### 3.4. Final Design

**Figure 8:**
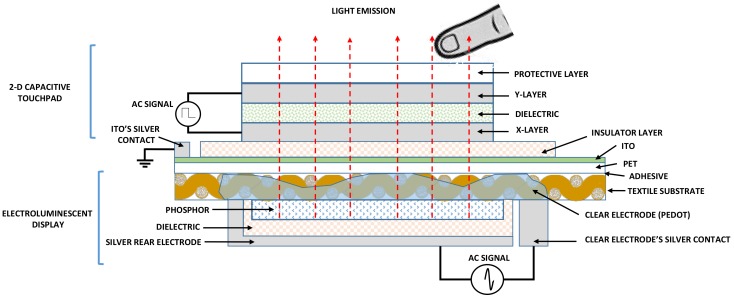
ELD + 2D touchpad architecture with ITO EMI shield. ELD: electroluminescent display; ITO: indium tin oxide; EMI: electromagnetic interferences.

**Figure 9:**
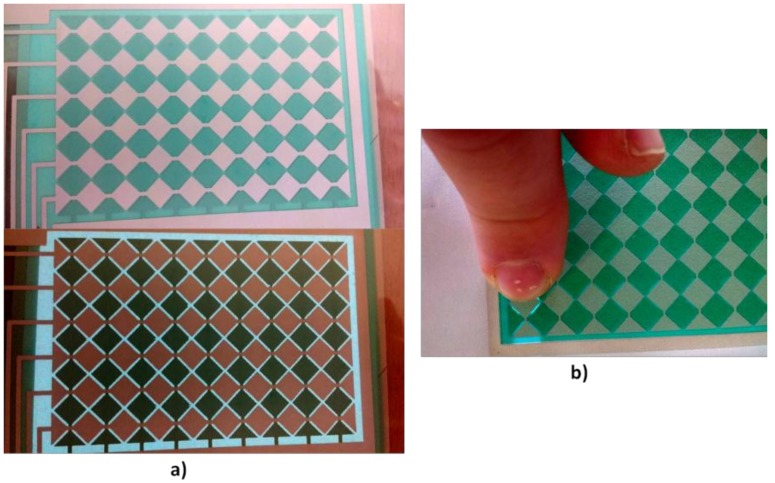
2D touchpad with ELD on and off (**a**). Redesign in order to turn on only the zone that has been touched (**b**). The design has been manufactured with Fabric_A.

A final design with an EMI shield was made as can be seen in Figure 8. This device allows to turn ELD on or off from the control electronics. A later version, in which the ELD has been redesigned so that only the zone that has been touched is turned on, has been made. (Figure 9).

## 4. Conclusions

A novel solution that combines a touchpad 2D with an electroluminescent (EL) matrix has been presented, using screen-printing technique on a textile substrate. The integration of a 2D touch sensor with an electroluminescent display is not directly due to the electromagnetic fields emitted by the ELD, so an EMI shield must be incorporated into the assembly, which makes it difficult, but not impossible, to translate it into the industrial field. The application of the ELD on textile reduces considerably the luminosity compared to using transparent substrate based on ITO, used as reference. Two solutions have been presented as alternatives to improve the luminosity, the ELD on a transparent substrate based on ITO and of polyurethane with PEDOT:PSS like emitting electrode and later to anchor it to the textile. However, in those cases in which the integration of an ITO film is not possible, and there are no excessive luminosity requirements, the full printed presented solution can be considered, taking into account the limitations previously explained. The entire screen-printing process adds a maximum of 200 μm to the textile, so it remains flexible, and the inks used are flexible, so there is no breakage when working with the material. Regarding the fabrics studied, all of them have a similar behaviour with the 2D sensor, but the highest luminous efficiency is obtained with Fabric_A. The developed equipment allows feedback with the user in the different applications of this type of system

## Appendix A

**Table A1:** Characteristics of the different fabrics used.

Fabric	Weft Density (Thread/cm)	Warp Density (Thread/cm)	Ligament	Grammage (g/m^2^)	Weft Material	Warp Material	Thickness (µm)
(A) Mediatex	25	40	Taffeta	115	Polyester	Polyester	110
(B) 65% Pol/35% Cot	30	46	Taffeta	110	Cotton	Polyester	200
(C) 100% Cotton	30	30	Taffeta	120	Cotton	Cotton	190
(D) 100% Cotton Waterproof	34	45	Taffeta	120	Cotton	Cotton	130

**Table A2:** Light characteristics of the different samples used.

pe	Substrate	Clear Conductor	lux	CIE 1931 Standard				Light Trans
				x	y	z	Dominant	
A	PET	ITO	217	0.3294	0.3745	0.2961	549.59	79%
B	Fabric_A	PEDOT:PSS	74.1	0.3195	0.3553	0.3253	514.31	27%
C	Fabric_B	PEDOT:PSS	11.3	0.3479	0.3428	0.3093	583.37	14%
D	Fabric_C	PEDOT:PSS	11.2	0.3059	0.3112	0.3829	480.36	32%
E	Fabric_D	PEDOT:PSS	29.2	0.3084	0.3374	0.3542	493.81	30%
F	Inspire 2370	PEDOT:PSS	170	0.2821	0.3328	0.3851	491.58	49%
G	Fabric_C	PEDOT:PSS + TRITON	15	0.2764	0.2503	0.4733	465.84	33%
H	Fabric_C	PEDOT:PSS + GLYCEROL	14	0.2701	0.2529	0.4770	471.12	32%
I	Fabric_C	PEDOT:PSS + GLYCEROL + TRITON	10	0.2813	0.2470	0.4717	457.22	33%
